# On the Logic of a Prior Based Statistical Mechanics of Polydisperse Systems: The Case of Binary Mixtures

**DOI:** 10.3390/e21060599

**Published:** 2019-06-16

**Authors:** Fabien Paillusson

**Affiliations:** School of Mathematics and Physics, University of Lincoln, Lincoln LN6 7TS, UK; fpaillusson@lincoln.ac.uk

**Keywords:** mixtures, entropy, polydispersity, binomial distribution

## Abstract

Most undergraduate students who have followed a thermodynamics course would have been asked to evaluate the volume occupied by one mole of air under standard conditions of pressure and temperature. However, what is this task exactly referring to? If air is to be regarded as a mixture, under what circumstances can this mixture be considered as comprising only one component called “air” in classical statistical mechanics? Furthermore, following the paradigmatic Gibbs’ mixing thought experiment, if one mixes air from a container with air from another container, all other things being equal, should there be a change in entropy? The present paper addresses these questions by developing a prior-based statistical mechanics framework to characterise binary mixtures’ composition realisations and their effect on thermodynamic free energies and entropies. It is found that (a) there exist circumstances for which an ideal binary mixture is thermodynamically equivalent to a single component ideal gas and (b) even when mixing two substances identical in their underlying composition, entropy increase does occur for finite size systems. The nature of the contributions to this increase is then discussed.

## 1. Introduction

Whether one thinks of ubiquitous substances such as air or drinking water or more specific fluids such as petroleum or milk, they all count as multicomponent systems, i.e., they all comprise more than one identifiable type of constituent. Furthermore, following early commentaries (see [1] and references therein) on Gibbs’ original work [2,3] on mixtures, the latter are thought to play a key role in the—quantum [4,5,6,7,8,9,10,11,12,13] or classical [14,15,16,17,18,19,20,21,22,23,24,25]—foundations of classical statistical mechanics; through the (in)famous *Gibbs paradox*. Somewhat surprisingly then, the vast majority of statistical mechanics textbooks covers principally single component systems. Mixtures are usually treated but as an exception to the identical particle paradigm. The reasons for this lack of visibility of multicomponent systems, despite their omnipresence in natural and artificial settings, are often rather elusive so that we could be left with—wrongly—attributing motives to their authors ranging from “*mixtures do not matter as much as we think*” to “*mixtures are too complicated to treat in all their details anyways*”. To be sure, if phase behaviour is to be considered in models of mixtures with interacting constituents, then characterising these phases and their occurrences is indeed a much more complicated problem than when looking at single component systems, as illustrated in relatively recent works [26,27]. Gibbs originally developed the notions of grand (and petit) canonical ensembles to elucidate the statistical thermodynamics of systems with varying particle numbers, including mixing problems [3]. More recently, the entropy of mixtures and Gibbs’ paradoxes were revisited within a more contemporary framework involving probabilities and particle exchanges protocols equivalent to the grand canonical ensemble for non-interacting systems [28]. However, for finite size systems, the grand canonical ensemble may not always give the same result as the canonical ensemble and, in practice, many mixing scenarios do not involve any external reservoir with which to exchange particles. In addition, we will see that, even in absence of interaction between constituents, it is arguable that the very nature of a polydisperse system in the canonical ensemble requires additional care in its statistical mechanics treatment which impacts the finite size expected canonical free energy and entropy measures. The purpose of this paper is therefore two-fold: (1) to develop a mathematically and conceptually consistent statistical mechanics treatment of mixtures in the canonical ensemble and (2) to derive general finite size expressions for the canonical measures of free energy and entropy. From these expressions, we will then show that there are circumstances for which a mixture, in the canonical ensemble, becomes equivalent to a single component system thus partially justifying the apparent lack of coverage in the literature. In what follows, we will focus on the statistical mechanics of binary mixtures to illustrate the kind of difficulties that can already emerge at this rather simple level of polydispersity. The article is organised as follows: Section 2 reminds the reader of the classical textbook treatment of binary mixtures in the canonical ensemble. Section 3 introduces a heuristic generalisation of the textbook treatment developed in Section 2 and discusses some of its caveats. Section 4 proposes a general, prior based, statistical mechanics framework for binary mixtures and discusses its consequences. Section 5 looks at the problem of mixing between two mixtures with different compositions and identifies the various contributions to the entropy change so as provide a specific definition of mixing entropy. Finally, Section 6 discusses further perspectives and Section 7 presents conclusions.

## 2. Textbook Treatment of Binary Mixtures

The textbook treatment of binary mixtures of non-interacting particles with Hamiltonian *H* usually considers a system with a total of *N* particles with $N_{1}$ particles of type 1 and mass $m_{1}$ and $N_{2}=N-N_{1}$ particles of type 2 and mass $m_{2}$, different from type 1, confined in a box of volume *V* and maintained at a temperature $T={\left(k_{B}\beta \right)}^{-1}$. Following the 1/n! prescription for any *n* identical particles in the system, the classical canonical partition function $Q(N,N_{1},\beta ,V)$ of the system then reads (e.g., in Refs. [8,9,11]):

(1) $$Q\left(N,N_{1},\beta ,V\right)=\frac{1}{N_{1}!\left(N-N_{1}\right)!}\int_{\mathrm{phase}\;\mathrm{space}}\frac{\prod_{i=1}^{N}d^{3}r_{i}d^{3}p_{i}}{h^{3N}}e^{-\beta H},$$

where *h*—usually taken as the Planck constant—is a quantity with the dimension of an action and the Hamiltonian *H* reads

(2) $$H=U_{\mathrm{box}}\left({\left\{{\vec{r}}_{i}\right\}}_{i=1,\ldots ,N}\right)+\sum_{i=1}^{N_{1}}\frac{{\vec{p}}_{i}^{2}}{2m_{1}}+\sum_{i=1}^{N-N_{1}}\frac{{\vec{p}}_{i}^{2}}{2m_{2}},$$

where $U_{\mathrm{box}}\left({\left\{{\vec{r}}_{i}\right\}}_{i=1,\ldots ,N}\right)$ is infinite if any particle goes outside the bounding box and is zero otherwise. Upon choosing, as is often done, $N_{1}=N/2$, it follows that:

(3) $$Q\left(N,N_{1}=N/2,\beta ,V\right)=\frac{1}{N_{1}!\left(N-N_{1}\right)!}\frac{V^{N}}{\Lambda_{1}^{3N_{1}}\Lambda_{2}^{3(N-N_{1})}}=\frac{1}{\left(N/2\right){!}^{2}}\frac{V^{N}}{\Lambda_{1}^{3N/2}\Lambda_{2}^{3N/2}},$$

where $\Lambda_{i}\equiv h\sqrt{\beta /(2\pi m_{i})}$ is the thermal wavelength of species *i*. Finally, applying the Stirling approximation lnN!≈NlnN−N, one finds for sufficiently large *N* the free energy

(4) $$\beta F\left(N,N_{1}=N/2,V,\beta \right)≃-N\ln2-N+\frac{N}{2}\ln\left(\rho \Lambda_{1}^{3}\right)+\frac{N}{2}\ln\left(\rho \Lambda_{2}^{3}\right),$$

where ρ≡N/V. In Equation (4), the second and third terms can be interpreted as the free energy that a gas of N/2 identical particles of respectively type 1 and 2 would have, had they been separated in equal sized compartments of volume V/2. Given that F=U−TS and that *T* does not vary, it is then rather timely to introduce the mixing entropy $\Delta S_{mix}$ as being:

(5) $$\frac{\Delta S_{mix}}{k_{B}}\equiv \beta F\left(N_{1}=N/2,\beta ,V/2\right)+\beta F\left(N_{2}=N/2,\beta ,V/2\right)-\beta F\left(N,N_{1}=N/2,\beta ,V\right)=N\ln2.$$

The entropy gain of $k_{B}\ln2$ per particle found in Equation (5) upon mixing is the well known entropy of mixing attributable to the difference in identity between the two species 1 and 2. Technically speaking, this difference in identity is betrayed by the factor ${\left(N/2!\right)}^{-2}$ in Equation (3), instead of the usual 1/N! expected for identical particles. However, it has been argued that interpreting the $Nk_{B}\ln2$ entropy change as stemming from this combinatorial factors was in fact misleading [11]. We will come back to this issue in Section 5.

## 3. Heuristic Generalisation

One of the issues with what was presented above as the “textbook” derivation is that it may appear somewhat artificial in a general context. For example, in the case where $N_{1}=N/2$, it follows that *N* ought to be an even number as there cannot be a fractional number of particles. In addition, even in absence of fractional particle numbers, how are we to choose the species upon adding a single additional particle to the overall system? These questions become more and more inevitable as the kind of mixtures one wants to look at becomes more complex with, e.g., $N_{1}=N/\sqrt{2}$ or when considering more than two species or even infinitely many.

One possible way of tackling these issues is to interpret the composition of a mixture through the *probability* p∈[0,1] for a particle in the box to be of type 1 and then recast $N_{1}$ as $N_{1}\equiv Np$. Since *p* is a probability, the product Np need not be an integer and each new particle added to the mixture will be of type 1 with probability *p* and type 2 with probability 1−p. With this new prescription, Equation (3) can be rewritten as follows:

(6) $$Q\left(N,p,\beta ,V\right)=\frac{1}{\Gamma (Np+1)\Gamma (N(1-p)+1)}\frac{V^{N}}{\Lambda_{1}^{3Np}\Lambda_{2}^{3N(1-p)}},$$

where $\Gamma \left(x+1\right)\equiv \int_{0}^{+\infty }dy\;y^{x}e^{-y}$ is the Euler gamma function which generalises the factorial function to the reals. For large values of *x*, one can use a saddle point approximation and finds that Γ(x+1) satisfies the Stirling approximation Γ(x+1)≃xlnx−x. Thus, in the large *N* limit, one finds for the free energy

(7) $$\beta F\left(N,p,\beta ,V\right)≃Np\ln\left(\rho \Lambda_{1}^{3}\right)+N\left(1-p\right)\ln\left(\rho \Lambda_{2}^{3}\right)-N-Ns\left(p\right),$$

where s(p)≡−plnp−(1−p)ln(1−p). Although some authors [19,26] refer to s(p) as the mixing entropy, we follow [29] and consider that the mixing entropy denomination should be left for unambiguously prepared mixing scenarios and their corresponding entropy *variations* and therefore refer to s(p), appearing in the equilibrium free energy of a mixture, as its *composition entropy*. For the reader who is more mathematically inclined, in the case of a Bernouilli random variable with probability *p*, s(p) is also called the *binary entropy* [30].

It can be noted that Equation (7) can be rewritten, without any explicit reference to different species, as follows:

(8) $$\beta F\left(N,p,\beta ,V\right)≃N\left(\ln\left(\rho {\tilde{\Lambda }}^{3}\right)-1\right)-Ns\left(p\right),$$

where $\tilde{\Lambda }\equiv h\sqrt{\beta /(2\pi \tilde{m})}$ and $\tilde{m}\equiv m_{1}^{p}m_{2}^{1-p}$ is the weighted geometric mean of the masses of the species 1 and 2. One important consequence of Equation (8) is that, at *fixed* composition, both $\tilde{\Lambda }$ and s(p) have fixed values and do not contribute to the thermodynamic properties of the system and therefore the non-interacting binary mixture is thermodynamically equivalent to a system of *N* effective identical particles [22].

**Figure 1:**
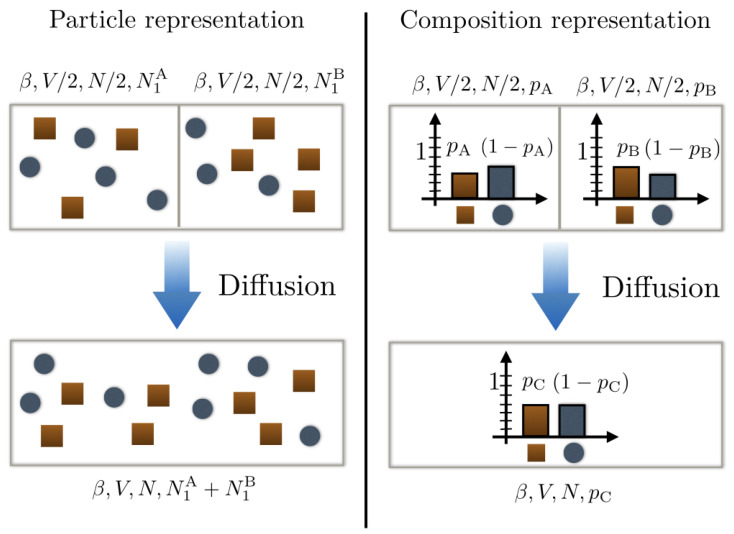
Schematic representation of a mixing scenario between two different substances. The top left panel uses a particle representation with type 1 particles as dark orange squares and type 2 particles as blue disks. We see that the compositions of substances A and B are different. This is further illustrated in representing their underlying probability distribution on the top right panel. Upon mixing the bottom panels, they form a new composition C that is a priori different from A and B.

Suppose now that we have two binary mixtures each comprising species 1 and 2 but with different compositions: mixture A with probability $p_{A}$ for a particle of the mixture to be of type 1 and mixture B with probability $p_{B}$ for a particle of the mixture to be of type 2. Each mixture contains N/2 particles and is initially confined in a box of volume V/2 (cf. Figure 1).

They are then mixed together in a box of volume *V*: what is the entropy change in the mixture? To address this question, we need first to determine the composition of the new mixture C obtained after mixing. In the absence of chemical reactions, the conservation of particle number compels us to ascribe the probability $p_{C}=\left(p_{A}+p_{B}\right)/2$. The entropy change upon mixing reads then

(9) $$\frac{\Delta S_{mix}}{k_{B}}=\beta F\left(N/2,p_{A},\beta ,V/2\right)+\beta F\left(N/2,p_{B},\beta ,V/2\right)-\beta F\left(N,p_{C},\beta ,V\right)=D_{\mathrm{JS}}\left(p_{A}|p_{B}\right),$$

where

(10) $$D_{\mathrm{JS}}\left(p_{A}|p_{B}\right)=\frac{1}{2}\sum_{i=A.B}\left[p_{i}\ln\left(\frac{2p_{i}}{p_{A}+p_{B}}\right)+\left(1-p_{i}\right)\ln\left(\frac{2(1-p_{i})}{\left(1-p_{A}\right)+\left(1-p_{B}\right)}\right)\right].$$

is the *Jensen–Shannon divergence*. Two features of $D_{\mathrm{JS}}$ worth mentioning is that it is positive definite and bounded from above by ln2 and its square root is a distance between probability distributions [31]. One can verify for example that if $p_{A}=0$ and $p_{B}=1$, then $D_{\mathrm{JS}}=\ln2$ thus retrieving Equation (5).

In spite of the generalisation of the textbook derivation to any binary mixture and the physical insights gained from adopting a *composition as probability* paradigm, the approach developed above suffers from a few mathematical and conceptual problems which need to be addressed:

- The heuristic approach used in this section starts off by directly generalising Equation (3) into Equation (6). Mathematically, it cannot start from Equation (1) as, in general, it could involve a fractional number of phase space integrals over particles of type 1 or 2, respectively. Thus, the current approach cannot be used as a basis to devise a mathematically rigorous composition-probability-based statistical mechanics of mixtures.
- Equation (6) made use of the Euler Gamma function to replace the more traditionally accepted n! terms at the denominator so as to account for Np not being an integer. While mathematically this may be fine, it is not justified within statistical mechanics itself and, indeed, as the first point was being raised, it is hardly so, as one could have a fractional number of phase space integrals.
- Problems arise with Np as well. What does it mean to switch from $N_{1}$ (an integer) to Np (not necessarily an integer) in the canonical ensemble? If *p* is a probability for a particle to be of type 1, then the particle type is a Bernouilli random variable t={1,2} such that p(t=1)=p and p(t=2)=1−p. Suppose we now model a mixture as a collection ${\left\{t_{i}\right\}}_{i=1,\ldots ,N}$ of *N* independent such random variables. This leads us to define the random variable $N_{1}\equiv \sum_{i=1}^{N}\left(-t_{i}+2\right)$ corresponding to the number of particles of type 1 in the system. If we denote 〈〈·〉〉, from the composition average, $\langle \langle N_{1}\rangle \rangle =Np$ comes. Thus, by substituting $N_{1}$ with Np in Equation (3), one effectively replaces an integral number of particles by a real positive expectation value. Conceptually, this poses a problem, at least in principle, as any given individual mixture will only ever have an integer number of particles usually close to, but different from, Np.

It is the aim of the following sections to devise a rigorous statistical mechanics framework of binary mixtures for which the composition is interpreted as an a priori probability distribution with probability *p* for any given particle to be of type 1 (and probability (1−p) for a particle to be of type 2). Only once such a framework has been laid out can we hope to delineate the range of validity, if any, of the results summarised in Equations (7)–(9) and the approximate reasoning used to derive them.

## 4. Prior Based Statistical Mechanics of a Binary Mixture

We now consider the simple case of the composition of a binary mixture modelled as a collection ${\left\{t_{i}\right\}}_{i=1,\ldots ,N}$ of *N* independent Bernouilli random variables with $p(t_{i}=1)=p$ and $p(t_{i}=2)=1-p$. Denoting $N_{1}$ as the random variable for the number of particles of type 1 among *N*, the probability $P(N_{1}=N_{1}|N,p)$ for it to be equal to a set integer value $N_{1}$ follows a Binomial distribution $B_{N,p}\left(N_{1}\right)$ defined by:

(11) $$P\left(N_{1}=N_{1}|N,p\right)=B_{N,p}\left(N_{1}\right)\equiv \frac{N!}{N_{1}!\left(N-N_{1}\right)!}p^{N_{1}}{\left(1-p\right)}^{N-N_{1}}.$$

Given the status of random variable of $N_{1}$, it means that the set of values it can take corresponds to a set of *different* possible realisations of the *same* mixture. As a side note, in models with interacting components, the fact that there can be multiple realisations of the same mixture (as characterised by an a priori probability distribution) translates into using random matrices to set the interaction strengths between constituents [27].

From Equation (11), we see that

(12) $$\frac{1}{N_{1}!\left(N-N_{1}\right)!}=\frac{B_{N,p}\left(N_{1}\right)}{N!p^{N_{1}}{\left(1-p\right)}^{N-N_{1}}}.$$

Now, for any given mixture realisation, both *N* and $N_{1}$ are fixed and the framework of the canonical ensemble applies, including Equation (3). We can then substitute $1/(N_{1}!\left(N-N_{1}\right)!)$ by its expression in Equation (12) and get:

(13) $$Q\left(N,N_{1}=N_{1},\beta ,V\right)=\frac{V^{N}}{\Lambda_{1}^{3N_{1}}\Lambda_{2}^{3(N-N_{1})}}\frac{B_{N,p}\left(N_{1}\right)}{N!p^{N_{1}}{\left(1-p\right)}^{N-N_{1}}}.$$

We seek a definition of the free energy of a mixture that would be realisation-independent. This is because repeating an experiment with a given mixture—characterised by prior composition probability—likely involves different realisations of its composition. To this end, we denote $F\left(N,p,V,\beta \right)\equiv \langle \langle -k_{B}T\ln Q\left(N,N_{1},\beta ,V\right)\rangle \rangle$ the canonical free energy averaged over realisations of $N_{1}$. After a bit of algebra and using the fact that $\langle \langle N_{1}\rangle \rangle =Np$, we get

(14) $$\beta F\left(N,p,V,\beta \right)=-N\ln V+N\ln{\tilde{\Lambda }}^{3}+\ln N!-Ns\left(p\right)+H\left(B_{N,p},B_{N,p}\right),$$

where s(p) and $\tilde{\Lambda }$ are as introduced in Equations (7) and (8), respectively, and $H\left(f,g\right)\equiv -\sum_{N_{1}=0}^{N}f\left(N_{1}\right)\ln g\left(N_{1}\right)$ making then $H(B_{N,p},B_{N,p})$ interpretable as the *realisation entropy* of the mixture. There does not exist any exact closed form expression for $H(B_{N,p}|B_{N,p})$ [32] but for *N* sufficiently large $H\left(B_{N,p},B_{N,p}\right)∼\left(1/2\right)\ln\left(2\pi eNp\left(1-p\right)\right)$ (see Appendix A) so that, in the large *N* limit, Equation (14) is equivalent to Equation (8), thus justifying more rigorously its validity.

A few remarks are in order regarding Equation (14):

- The large *N* limit leading to Equation (8) in the heuristic derivation (Section 3) implies that both Np and N(1−p) should be sufficiently large for the Stirling approximation to hold, which can require a very large *N* if *p* is either very small or very close to unity. On the contrary, Equation (14) converges rather quickly with *N* to the asymptotic form Equation (8) *especially* when *p* is either very small or very close to unity. This can be explained by remarking that, if 1−p is very small and *N* finite, any realisation will most likely have very few, if any, particles of type 2. As a consequence, the realisation entropy will be closer to zero in magnitude as there is less uncertainty in the composition realisations. Therefore, although the heuristic derivation leads to the asymptotic form Equation (8), it incorrectly—given that Equation (14) has stronger mathematical and conceptual foundations—predicts the composition for which the asymptotic regime is reached the fastest.
- For finite *N*, the realisation entropy term in Equation (14) actually *decreases* the entropy estimate given by the first four terms in the r.h.s of Equation (14) as it contributes *negatively* to the entropy of the system. This can be understood by adopting a “surprise” interpretation of entropy. The last two terms of Equation (14) can then be interpreted as the average surprise to have $N_{1}$ type 1 particles in the mixture. On the one hand, the Ns(p) contribution to entropy stems from an estimation of the surprise for a given realisation $N_{1}$ as being $N_{1}\ln p+\left(N-N_{1}\right)\ln\left(1-p\right)$. This would be exact if one were to either assign a particular order to the particles or if one were to repeat single-particles experiments *N* times, where the identity of the particle for each try is obtained from the underlying probability distribution of the Bernouilli variable t, and add-up the observed individual surprises [22]. On another hand, the probability to have $N_{1}$ particles of type 1 among *N* is $B_{N,p}\left(N_{1}\right)$ and the corresponding surprise is $-\ln B_{N,p}\left(N_{1}\right)$. The difference between the two gives us the *relative surprise*$-\ln\frac{p^{N_{1}}{\left(1-p\right)}^{N-N_{1}}}{B_{N,p}\left(N_{1}\right)}$ which captures the contribution to entropy owing to composition.

Finally, we note that Sollich et al. [26,33] have proposed a strategy based on a moment-description of thermodynamic quantities for sufficiently large *N* to tackle the fact that a mixture composition of a single finite system is but a realisation of some underlying prior composition. This strategy differs in spirit from the one developed in the present paper in that it aims at performing a dimensional reduction of the free energy landscape by using a dependence of the free energy in the moments (ideally a small number of them) of the feature used to characterise the composition (e.g., size. mass, etc...) to obtain insights on the phase behaviour of mixtures. This objective is currently beyond the scope of the present paper.

## 5. Identifying the Gibbs Mixing Entropy

In this section, we consider a situation analogous to the one described in Figure 1 whereby N/2 particles (*N* being even) of a mixture A initially confined in a volume V/2 mix with N/2 particles of a mixture B initially confined in a volume V/2. Mixtures A and B only comprise type 1 and 2 particles but each particle identity is modelled with different random variables depending on the mixture it belongs to: $t^{A}$ with $p\left(t^{A}=1\right)=p_{A}$ and $t^{B}$ with $p\left(t^{B}=1\right)=p_{B}$. Following the theory developed in Section 4, we model the composition of mixture A (resp. B) as a collection of N/2 independent Bernouilli random variables ${\left\{t_{i}^{A}\right\}}_{i=1,\ldots ,N/2}$ (resp. ${\left\{t_{i}^{B}\right\}}_{i=1,\ldots ,N/2}$) and denote $N_{1}^{A}$ (resp. $N_{1}^{B}$) the random variable giving the number of type 1 particles in mixture A (resp. mixture B). $N_{1}^{A}$ and $N_{1}^{B}$ both follow a Binomial distribution and therefore, prior to mixing, the free energy of each mixture—for a given realisation—follows from Equation (12):

(15) $$\beta F_{A/B}\left(N/2,N_{1}^{A/B}=N_{1}^{A/B},\beta ,V/2\right)=-\ln\left[\frac{V^{\frac{N}{2}}}{\Lambda_{1}^{3N_{1}^{A/B}}\Lambda_{2}^{3(N/2-N_{1}^{A/B})}}\frac{B_{\frac{N}{2},p_{A/B}}\left(N_{1}^{A/B}\right)}{\frac{N}{2}!p_{A/B}^{N_{1}^{A/B}}{\left(1-p_{A/B}\right)}^{N/2-N_{1}^{A/B}}}\right].$$

Upon mixing, the A and B mixtures will form a new mixture C with a composition emerging from the constraints during the diffusion process [29]. In the absence of chemical reactions, the number $N_{1}^{C}$ of particles of type 1, once mixing has occurred, is a random variable satisfying:

(16) $$N_{1}^{C}\equiv N_{1}^{A}+N_{1}^{B},$$

and

(17) $$\langle \langle N_{1}^{C}\rangle \rangle =\frac{N}{2}\left(p_{A}+p_{B}\right)=Np_{C}.$$

We note that Equation (17) justifies the approach used for mixture C within the heuristic approach described in Section 3. If we want to know what is the probability for having $N_{1}^{C}=N_{1}^{C}$, then we have:

(18) $$\begin{array}{ccc} P\left(N_{1}^{C}=N_{1}^{C}|N,p_{A},p_{B}\right)=P_{N,p_{A},p_{B}}\left(N_{1}^{C}\right) & \equiv & \sum_{N_{1}^{A}=0}^{N/2}\sum_{N_{1}^{B}=0}^{N/2}B_{\frac{N}{2},p_{A}}\left(N_{1}^{A}\right)B_{\frac{N}{2},p_{B}}\left(N_{1}^{B}\right)\delta_{N_{1}^{C},N_{1}^{A}+N_{1}^{B}} \end{array}$$

(19) $$\begin{array}{cc} \;= & \sum_{N_{1}^{A}=0}^{N_{1}^{C}}B_{\frac{N}{2},p_{A}}\left(N_{1}^{A}\right)B_{\frac{N}{2},p_{B}}\left(N_{1}^{C}-N_{1}^{A}\right), \end{array}$$

where $\delta_{i,j}=1$ if i=j, and zero otherwise, is the Kronecker delta function. If $p_{A}=p_{B}$, then $P_{N,p_{A},p_{B}}\left(N_{1}^{C}\right)=B_{N,p_{A}}\left(N_{1}^{C}\right)$. However, there is no known closed form expression for $P_{N,p_{A},p_{B}}\left(N_{1}^{C}\right)$ when $p_{A}\neq p_{B}$ so if one wants to estimate its values it has to be done either numerically by carrying out the whole summation or by using an approximate expression [30].

The canonical free energy of the final state of the system for a given realisation $N_{1}^{C}$ can be written from the form of the canonical partition function in Equation (13):

(20) $$F_{C}\left(N,N_{1}^{C}=N_{1}^{C},\beta ,V\right)=-\ln\left[\frac{V^{N}}{\Lambda_{1}^{3N_{1}^{C}}\Lambda_{2}^{3(N-N_{1}^{C})}}\frac{B_{N,p_{C}}\left(N_{1}^{C}\right)}{N!p_{C}^{N_{1}^{C}}{\left(1-p_{C}\right)}^{N-N_{1}^{C}}}\right].$$

The difference with Equation (13), however, is that, in Equation (20), the probability distribution $B_{N,p_{C}}\left(N_{1}^{C}\right)$ is in general not equal to the actual probability distribution (given in Equation (19)) for obtaining $N_{1}^{C}$ type 1 particles after the mixing of substances A and B. We will see in what follows that, as long as $B_{N,p_{C}}\left(N_{1}^{C}\right)$ has the same support as $P(N_{1}^{C}=N_{1}^{C}|N,p_{A},p_{B}),$ this problem can be overcome.

For a given realisation of mixtures A and B composition, we can now obtain the entropy variation $\Delta S_{mix}$ upon mixing as being of the two gases from $\Delta S_{mix}/k_{B}=\beta F_{A}\left(N/2,N_{1}^{A},\beta ,V\right)+\beta F_{B}\left(N/2,N_{1}^{B},\beta ,V\right)-\beta F_{C}\left(N,N_{1}^{C},\beta ,V\right)$. For each realisation, a different entropy variation can be be found in principle. Like in Section 4, a realisation-free entropy change $\Delta S_{mix}\equiv \langle \langle \Delta S_{mix}\rangle \rangle$ can be sought by averaging over composition realisations of substances A and B. We get (see Appendix B):

(21) $$\frac{\Delta S_{mix}}{k_{B}}=\underset{\mathrm{partitioning}\;\mathrm{entropy}}{\underset{︸}{\ln\left(\frac{2^{N}{\left(\left(N/2\right)!\right)}^{2}}{N!}\right)}}+N\underset{\mathrm{square}\;\mathrm{metric}}{\underset{︸}{D_{JS}\left(p_{A}|p_{B}\right)}}+\underset{\mathrm{realisation}\;\mathrm{entropy}}{\underset{︸}{\sum_{i=A,B}H\left(B_{\frac{N}{2},p_{i}},B_{\frac{N}{2},p_{i}}\right)-H\left(P_{N,p_{A},p_{B}},B_{N,p_{C}}\right)}}.$$

In Equation (21), we have identified three different contributions to the entropy change upon mixing substances A and B that are worth commenting on:

- **Partitioning**: The first term on the r.h.s of Equation (21) corresponds to the entropy change owing to having removed a partition between the initial compartments or, said differently, results from the more numerous partitioning possibilities offered when the whole volume *V* can be explored instead of V/2. Indeed, when each particle can freely roam in the boxe of volume *V*, imagining a virtual division splitting the box into two equal half will lead each particle to be either on one side or the other of the virtual dividing wall. There are then $2^{N}$ possibilities. When the dividing wall switches from virtual to real, and N/2 particles have to be on each side, the number of ways of realising this partitioning is $N!/{\left(\left(N/2\right)!\right)}^{2}$. The corresponding entropy change is therefore larger than zero for any finite *N*.
- **Composition distance**: The second term on the r.h.s of Equation (21) has already been discussed in Section 4 and corresponds to a contribution to the entropy variation stemming from how different are the compositions of mixtures A and B when characterised by underlying probability distributions for particle identities. Since $D_{JS}\left(p_{A}|p_{B}\right)$ is a square metric, it becomes strictly zero when the a priori compositions are identical.
- **Composition realisation**: The third and last term identified on the r.h.s of Equation (21) corresponds to the entropy variation owing to realisation considerations. It is worth noting that when the compositions are identical i.e., $p_{A}=p_{B}$ then $P_{N,p_{A},p_{B}}=B_{N,p_{A}}$ but $H\left(B_{N,p_{A}},B_{N,p_{A}}\right)\leq 2H\left(B_{\frac{N}{2},p_{A}},B_{\frac{N}{2},p_{A}}\right)$ because of the *submodular* character of Shannon’s entropy function [34]. As a result, the entropy variation due to composition realisations is larger than or equal to zero upon mixing even when substances have the same underlying composition.

When split into the three contributions identified in Equation (21), the reasons for an entropy increase upon mixing can be seen in a new light.

First, as noted in [11], conflating the presence of combinatorial terms in an entropy expression with an explicit role of particle identity can be misleading. Indeed, the partitioning entropy term has nothing to do with whether substances A and B are considered identical or not and is therefore not exclusive to the mixing of different substances. It will therefore appear in all mixing scenarios no matter what. This positive increase in entropy owing to partitioning can, at least in principle, be used to do meaningful work as exemplified with Szilard’s engine [35]. This positive increase in entropy also further supports the—usually dismissed—intuitive claim that, even when substances are identical, some form of mixing does indeed occur.

The realisation entropy contribution that is positive even when substances have an identical underlying composition can be understood by the fact that upon mixing information on the initial realisations of mixtures A and B has disappeared. Therefore, if one were to insert a dividing wall, even if it happens that there are N/2 particles on each side, the realised compositions on either sides of the wall will be differing from the initial ones, even if selective membrane is used. This difference in realisation can in principle be used to do work by adapting Szilard’s engine to incorporate a semi-permeable membrane. This conclusion would not follow, however, if the substances are identical in their underlying composition and $p_{A}$ is exactly equal to 0 or 1 since the consideration on realisations would then become meaningless.

It remains then the square metric contribution to entropy which is the only one to exactly vanish if substances are identical in their underlying composition and to measure in what sense substances A and B differ from each other in principle. For reasons detailed elsewhere [22], we argue that this contribution reflects best Gibbs’ original insights on the mixing of substances by diffusion [2] and shall therefore denote $\Delta S_{mix}^{\mathrm{Gibbs}}\equiv k_{B}ND_{JS}\left(p_{A}|p_{B}\right)$.

All of the above considerations with regard to Equation (21) hold for finite size systems. From a standard use of Stirling’s approximation, the first term on the r.h.s of Equation (21) vanishes in the thermodynamic limit. At first glance, it is nevertheless unclear how the $H(P_{N,p_{A},p_{B}},B_{N,p_{C}})$ behaves in the large *N* limit. It can be shown (Appendix A) that the term $H(P_{N,p_{A},p_{B}},B_{N,p_{C}})$ is at most of order ∼O(lnN) so that

(22) $$\Delta S_{mix}≃\Delta S_{mix}^{\mathrm{Gibbs}}=k_{B}ND_{JS}\left(p_{A}|p_{B}\right)$$

for large *N*.

## 6. Discussion

After having briefly presented what we called the textbook treatment of binary mixtures, we looked at a heuristic generalisation which is grounded in the idea that a size-independent definition of a substance necessitates the existence of a prior underlying probability distribution from which are drawn the particles identity. The heuristic formulation works by substituting $N_{1}$ the number of particles of type 1 by Np, *p* being the probability for an individual particle to be of type 1. Following some postulated extensions of the canonical ensemble framework, this led us to Equation (13) which enabled us to discuss how the thermodynamics of a binary mixture can then be equivalent to that of a system of identical particles, provided the composition is kept fixed. Acknowledging various caveats of the heuristic approach, we then developed a more rigorous approach fully compatible with traditional statistical mechanics. This new approach starts by taking seriously the idea of a prior probability distribution underlying the composition of a substance. If that is the case, then two different realisations of the same underlying distribution need not have the exact same composition. Having this in mind, it then conjectured that to obtain a realisation-independent thermodynamics of a binary mixture, one needs to consider the free energies averaged over realisations. This procedure enabled us to retrieve Equation (13) in the large *N* limit, thus validating the more intuitive approach used in Section 3. Incidentally, this further supports the idea that for dilute gases or substances at fixed composition, the actual polydisperse character of the substance bears no consequences on the thermodynamics of the system and it is enough to consider the system as comprising only—effective—identical particles. It is unclear whether this is purposeful or not, but this equivalence between a binary mixture and a single component system can partially justify—at least a posteriori—the predominance of single component systems in the literature available at undergraduate level.

Aside from the average free energy of a given mixture, the entropy variation upon mixing two different mixtures has also been studied within the new statistical mechanics framework proposed in this paper. It was found that the entropy of mixing comprised three physically different contributions: one owing to partitioning that is not exclusive to the mixing of different substances, one owing to a square distance between the underlying probability distributions for particles’ identity and a last one owing to realisation entropy differences. This last term is new and does not vanish when the underlying compositions of the two mixed substances are the same. Upon inspection, the second—metric based—term in Equation (21) is therefore the closest to the entropy of mixing, as discussed by Gibbs in [2]. Finally, we discussed the thermodynamic limit behaviour of Equation (21) and showed that it reduced to the Gibbs entropy (22).

It is worth noting that the proposed theory of binary mixtures relies on the assumption that the substances can be modelled as collections of independent random—identity—variables. In practice, any protocol which behaves the same way with any particle regardless of how many of a specific kind it has already processed, would give rise to a Binomial distribution for particle number of type 1 (resp. 2). However, there can also be non-independent collections of Bernouilli variables with $P_{N,p}\left(N_{1}\right)\neq B_{N,p}\left(N_{1}\right)$ (e.g., kinetic proof reading or active sorting) [36]. In such cases, the mathematical framework developed in these pages is still valid, but the asymptotic behaviours of average free energies need to be elucidated for each individual case. In addition, it is important to stress that, if the particles were to interact with each other, that would not necessarily mean that the particles’ identity random variables are not independent. Instead, particle interactions could simply be expressed through random matrices [27]. Therefore, one avenue of exploration would be to adapt it to interacting mixtures. Moreover, it is possible to see a parallel with the composition-realisation-based statistical mechanics developed in the present paper and averages over disorder in systems with quenched disorder (see e.g., [37]). This offers possibilities of cross-fertilisation between different branches of statistical mechanics. Now, if the realisation-based approach corresponds to averages over quenched disorder in other fields, it is tempting to draw a parallel between grand canonical ensemble-treatments of mixtures [28] and annealed disorder. The author is not aware of an equation equivalent to Equation (22) in the context of the grand canonical ensemble and this is another route worth exploring.

Finally, in Refs. [22,29], the heuristic approach was used to suggest that both discrete and continuous polydisperse systems were equivalent to a system of identical particles, provided the composition remains the same and a generalised version of Equation (9) was derived for mixtures of arbitrary number of components and composition. Whether these results can remain valid in a version of the present framework extended to more than two components remains to be determined.

## 7. Conclusions

In this article we proposed a theory to address the possibility of different realisations of a given composition and obtain realisation-independent free energies in the canonical ensemble. This lead us to find that (a) in addition to the composition entropy discussed in [22,29] a realisation entropy—vanishing in the thermodynamic limit—emerges in the expression of the free energy of a binary mixture, (b) for a fixed composition the thermodynamics of a binary mixture is equivalent to that of a system of identical particles, (c) the entropy change upon mixing two finite size identical substances has two positive contributions owing to firstly an increase in partitioning entropy and secondly a loss of information on substances’ realisations and (d) in the thermodynamic limit the mixing entropy is a square norm between the compositions of the substances being mixed. Further work remains to be done to extent this work to more complex mixtures.

## Appendix A. Limiting Behaviour of *H*(*P__N_,p_*A*_,p_*B*__*,*B_N,p_*C*__*)

We wish to determine the asymptotic behaviour of $H(P_{N,p_{A},p_{B}},B_{N,p_{C}})$ as *N* tends to infinity. To this end, we first note that $H(P_{N,p_{A},p_{B}},B_{N,p_{C}})\geq 0$ as it is the cross entropy over a discrete probability distribution. Next, we recall Jensen’s inequality [38], which, for a given random variable X, states that E[φ(X)]≥φ(E[X]), where E[·] stands for the expectation value. If we choose $B_{N,p_{C}}\left(N_{1}^{C}\right)/P_{N,p_{A},p_{B}}\left(N_{1}^{C}\right)$ for X and φ(x)=lnx, we get

(A1) $$E\left[\varphi \left(X\right)\right]=\sum_{N_{1}^{C}=0}^{N}P_{N,p_{A},p_{B}}\left(N_{1}^{C}\right)\ln\left(\frac{B_{N,p_{C}}\left(N_{1}^{C}\right)}{P_{N,p_{A},p_{B}}\left(N_{1}^{C}\right)}\right)\geq \ln\left[\sum_{N_{1}^{C}=0}^{N}B_{N,p_{C}}\right]=0,$$

which yields

(A2) $$H\left(P_{N,p_{A},p_{B}},B_{N,p_{C}}\right)\leq H\left(P_{N,p_{A},p_{B}},P_{N,p_{A},p_{B}}\right).$$

Note that Equation (A1) is valid because $B_{N,p_{C}}$ and $P_{N,p_{A},p_{B}}$ have the same support.

Next, we seek to evaluate the limiting behaviour of $H(P_{N,p_{A},p_{B}},P_{N,p_{A},p_{B}})$. We recall that $P_{N,p_{A},p_{B}}$ stands for the probability distribution of the sum of two binomial variables distributed according to $B_{\frac{N}{2},p_{A}}$ and $B_{\frac{N}{2},p_{B}}$, respectively. In the large *N* limit, the central limit theorem applies and we have

(A3) $$B_{\frac{N}{2},p_{A/B}}\left(N_{1}^{A/B}\right)∼N_{\frac{Np_{A/B}}{2},\frac{Np_{A/B}\left(1-p_{A/B}\right)}{2}}\left(N_{1}^{A/B}\right),$$

where

(A4) $$N_{\mu ,\sigma^{2}}\left(x\right)\equiv \frac{1}{\sqrt{2\pi \sigma^{2}}}e^{-\frac{{\left(x-\mu \right)}^{2}}{2\sigma^{2}}}.$$

Given that $N_{1}^{C}=N_{1}^{A}+N_{1}^{B}$, we can use Equation (A3) and consider $N_{1}^{C}$ as a sum of two normally distributed random variables. Since the sum of two normally distributed random variables with mean $\mu_{A}$ (resp. $\mu_{B}$) and variance $\sigma_{A}^{2}$ (resp. $\sigma_{B}^{2}$) is also normally distributed but with mean $\mu_{A}+\mu_{B}$ and variance $\sigma_{A}^{2}+\sigma_{B}^{2}$,

(A5) $$P_{N,p_{A},p_{B}}\left(N_{1}^{C}\right)∼N_{Np_{C},\frac{N}{2}\left(p_{A}\left(1-p_{A}\right)+p_{B}\left(1-p_{B}\right)\right)}\left(N_{1}^{C}\right).$$

is provided.

It is straightforward to show that

(A6) $$H\left(N_{\mu ,\sigma^{2}},N_{\mu ,\sigma^{2}}\right)=\frac{1}{2}\ln\left(2\pi e\sigma^{2}\right).$$

Thus, denoting $\sigma_{C}^{2}=N\left(p_{A}\left(1-p_{A}\right)+p_{B}\left(1-p_{B}\right)\right)/2$, we get that

(A7) $$H\left(P_{N,p_{A},p_{B}},P_{N,p_{A},p_{B}}\right)∼H\left(N_{Np_{C},\sigma_{C}^{2}},N_{Np_{C},\sigma_{C}^{2}}\right)∼O\left(\ln N\right),$$

which, when combined with Equation (A2), shows that $H(P_{N,p_{A},p_{B}},B_{N,p_{C}})$ is at most of order ∼O(lnN) in the large *N* limit.

## Appendix B. Deriving the Entropy Change Averaged over Compositions

In this section, we want to find an expression for the entropy variation upon mixing two substances averages over all possible realisations of their composition. We start by discussing more formally which average is taken. In our particular case of mixing, the average of any function $f(N_{1}^{A},N_{1}^{B})$ is defined as

(A8) $$\langle \langle f\left(N_{1}^{A},N_{1}^{B}\right)\rangle \rangle \equiv \sum_{N_{1}^{A}=0}^{N/2}\sum_{N_{1}^{B}=0}^{N/2}B_{\frac{N}{2},p_{A}}\left(N_{1}^{A}\right)B_{\frac{N}{2},p_{B}}\left(N_{1}^{B}\right)f\left(N_{1}^{A},N_{1}^{B}\right).$$

From the general definition above, two specific cases are worth looking at:

- If $f\left(N_{1}^{A},N_{1}^{B}\right)=f\left(N_{1}^{A/B}\right)$ i.e., only depends on one of the variables, then
  (A9) $$\langle \langle f\left(N_{1}^{A},N_{1}^{B}\right)\rangle \rangle =\sum_{N_{1}^{A/B}=0}^{N/2}B_{\frac{N}{2},p_{A/B}}\left(N_{1}^{A/B}\right)f\left(N_{1}^{A/B}\right).$$
- If $f\left(N_{1}^{A},N_{1}^{B}\right)=g\left(N_{1}^{A}+N_{1}^{B}\right)$, where g(x) is a function of a single variable, then
  (A10) $$\begin{array}{cc} \langle \langle f\left(N_{1}^{A},N_{1}^{B}\right)\rangle \rangle & =\sum_{N_{1}^{A}=0}^{N/2}\sum_{N_{1}^{B}=0}^{N/2}B_{\frac{N}{2},p_{A}}\left(N_{1}^{A}\right)B_{\frac{N}{2},p_{B}}\left(N_{1}^{B}\right)g\left(N_{1}^{A}+N_{1}^{B}\right) \end{array}$$
  (A11) $$\begin{array}{c} \;=\sum_{N_{1}^{C}=0}^{N}\sum_{N_{1}^{A}=0}^{N_{1}^{C}}B_{\frac{N}{2},p_{A}}\left(N_{1}^{A}\right)B_{\frac{N}{2},p_{B}}\left(N_{1}^{C}-N_{1}^{A}\right)g\left(N_{1}^{C}\right) \end{array}$$
  (A12) $$\begin{array}{c} \;=\sum_{N_{1}^{C}=0}^{N}P_{N,p_{A},p_{B}}\left(N_{1}^{C}\right)g\left(N_{1}^{C}\right). \end{array}$$

We are now equipped to look at the average of $\Delta S_{mix}/k_{B}=\beta F_{A}\left(N/2,N_{1}^{A},\beta ,V\right)+\beta F_{B}\left(N/2,N_{1}^{B},\beta ,V\right)-\beta F_{C}\left(N,N_{1}^{C},\beta ,V\right)$. To this end, we note that the first two free energies are functions of only one of the particle number and will therefore follow Equation (A10). This case is rather straightforward and gives an expression of the form (14):

(A13) $$\begin{array}{cc} \langle \langle \beta F_{A}\left(N/2,N_{1}^{A},\beta ,V\right)+\beta F_{B}\left(N/2,N_{1}^{B},\beta ,V\right)\rangle \rangle & =-N\ln\frac{V}{2}+\frac{N}{2}\left(\ln\left[{\left(\Lambda_{1}^{3}\right)}^{p_{A}}{\left(\Lambda_{2}^{3}\right)}^{1-p_{A}}\right]+\ln\left[{\left(\Lambda_{1}^{3}\right)}^{p_{B}}{\left(\Lambda_{2}^{3}\right)}^{1-p_{B}}\right]\right)\\ & +\ln{\left(\frac{N}{2}!\right)}^{2}-\frac{N}{2}\left(s\left(p_{A}\right)+s\left(p_{B}\right)\right)\\ & +H\left(B_{\frac{N}{2},p_{A}},B_{\frac{N}{2},p_{A}}\right)+H\left(B_{\frac{N}{2},p_{B}},B_{\frac{N}{2},p_{B}}\right). \end{array}$$

Next, the average of the free energy once mixing has occurred follows the case described in Equation (A12) and we get:

(A14) $$\begin{array}{cc} \langle \langle \beta F_{A}\left(N,N_{1}^{C},\beta ,V\right)\rangle \rangle & =-N\ln V+\langle \langle N_{1}^{C}\rangle \rangle \ln\Lambda_{1}^{3}+\langle \langle N-N_{1}^{C}\rangle \rangle \ln\Lambda_{2}^{3}+\ln N!\\ & -\langle \langle N_{1}^{C}\rangle \rangle \ln p_{C}-\langle \langle N-N_{1}^{C}\rangle \rangle \ln\left(1-p_{C}\right)+H\left(P_{N,p_{A},p_{B}},B_{N,p_{C}}\right). \end{array}$$

Substituting $\langle \langle N_{1}^{C}\rangle \rangle$ by $Np_{C}$ and subtracting Equation (A14) from (A13) then gives Equation (21).
